# Evolution of Parathyroid Hormone Receptor Family and Their Ligands in Vertebrate

**DOI:** 10.3389/fendo.2015.00028

**Published:** 2015-03-10

**Authors:** Jason S. W. On, Billy K. C. Chow, Leo T. O. Lee

**Affiliations:** ^1^School of Biological Sciences, The University of Hong Kong, Hong Kong, China

**Keywords:** parathyroid hormone, parathyroid hormone receptor, evolution, vertebrate, G protein-coupled receptor

## Abstract

The presence of the parathyroid hormones in vertebrates, including PTH, PTH-related peptide (PTHrP), and tuberoinfundibular peptide of 39 residues (TIP39), has been proposed to be the result of two rounds of whole genome duplication in the beginning of vertebrate diversification. Bioinformatics analyses, in particular chromosomal synteny study and the characterization of the PTH ligands and their receptors from various vertebrate species, provide evidence that strongly supports this hypothesis. In this mini-review, we summarize recent advances in studies regarding the molecular evolution and physiology of the PTH ligands and their receptors, with particular focus on non-mammalian vertebrates. In summary, the PTH family of peptides probably predates early vertebrate evolution, indicating a more ancient existence as well as a function of these peptides in invertebrates.

Although the physiological actions of parathyroid hormone family are well-documented in the literature, review about the molecular evolution of PTH ligands and their receptors are limited. Therefore, in this article, we reviewed recent studies about the PTH ligands and their receptors in different vertebrate species. We believe this mini-review can provide a better overview about the molecular evolution of this ligand and receptor family in vertebrate.

## Parathyroid Hormone Family

The parathyroid hormone peptide subfamily includes PTH, PTH-related peptide (PTHrP), and tuberoinfundibular peptide of 39 residues (TIP39, also known as PTH2). These hormones are encoded in separate genes but their mature peptides share significant sequence homology within the first 34 amino acids. In humans, PTH is highly expressed and is secreted by the parathyroid gland, but lower levels of its transcript can also be detected in the hypothalamus, pituitary, and thymus (1–3). PTH is an important regulator for body calcium homeostasis. In contrast to PTH, PTHrP is widely expressed in a broad spectrum of tissues, including central nervous system (4). The main function of PTHrP is the regulation of chondrocyte growth and differentiation in the growth plates of developing long bones (5). TIP39 shares a relatively lower level of sequence homology with PTH or PTHrP, but high resolution NMR studies suggest that it has a similar three-dimensional structure when compared to them (6). TIP39 is mainly synthesized in two brain regions, the subparafascicular area of the thalamus and the medial paralemniscal nucleus of the pons (7). Recent reports suggest that TIP39 is a neuroendocrine hormone that modulates several aspects of the stress response, as well as controls body temperature (8). In addition to PTH, PTHrP, and TIP39, there is a newly identified member of the family, PTH-like peptide (PTH-L), which is only present in non-mammalian species including *Xenopus*, teleosts, and chicken. *pth-l* gene structure is similar to that of the *pth gene*, but the mature peptide of PTH-L shares a higher level of sequence homology to PTHrP (9). The expression of PTH-L has been investigated in chicken and *Xenopus*. The peptide was found widely, but differentially expressed in various tissues of these organisms. Abundant PTH–L transcripts were detected in cartilage in chicken, and in brain, lung, and bone in *Xenopus laevis* (10). Although the physiological role has not yet been fully established, some reports have suggested that PTH-L in teleosts (Seabream) and *X. laevis* is the most potent calciotropic factor among all PTH peptides (10, 11).

## PTH Receptor Family

The importance of the PTH system as a regulator to control blood calcium levels was recognized early in the 20th century by observing the correlation between the parathyroid gland and tetany (12, 13). However, understanding of the physiological function of PTH was not substantially developed until the discovery of the human PTH1R in 1991 (14). PTH1R is also called the PTH/PTHrP receptor because of its equal binding affinities for both PTH and PTHrP. Subsequently, PTH2R, with a ligand preference for PTH over PTHrP, was identified in humans (15). However, further investigations suggested that TIP39 is the native ligand of PTH2R because of the robust activation of human PTH2R by TIP39 and the poor activation of rat PTH2R by PTH (16). In the last two decades, due to the success of different genome projects, paralogs and orthologs of PTHRs have been identified from various vertebrate species. These studies have also led to the discovery of an additional member of the receptor family, PTH3R, which is found only in non-mammalian vertebrates. The ligand specificity and structure of PTH3R resemble PTH1R more than PTH2R (17–19). In zebrafish, seabream, and chicken, PTH3R shows a stronger affinity for PTHrP than do other members of the PTH receptor family (17, 18, 20).

## Evolution of PTH and the PTHR Family

The PTHRs belong to the class B1 G protein-coupled receptors (GPCRs). It is widely believed that the paralogs of PTHR, *pth1r, pth2r*, and *pth3r* are evolved after two rounds of whole genome duplication (2WGD) (17, 21–23). Recent genome synteny comparison provided strong evidence to support this hypothesis (22). From teleosts to humans, PTHRs are found in three conserved loci. Interestingly, PTH2R and PTH3R are not found in birds and mammals, respectively. The genes for the PTHR cognate ligands (*pth, pthrp*, and *tip39*) are also located in three conserved loci and were likely generated through similar WGD events as their receptors. The *pth-l* gene is found in the fourth locus in chicken, which shares considerable synteny with the other three conserved loci of the *pth* gene family. Specifically in teleost, the third WGD generate extra copies of *pth* (*pth1* and *pth2*) and *pthrp* (*pthrpA* and *pthrpB*), but whether the copies share the synteny with PTH gene family remains to be investigated (11, 24).

The elephant shark was diverged at the time point just after the vertebrate 2WGD; therefore, its primitive genome provides insight into the evolution of the PTH and PTHR family (25, 26). Consistent with the “4:1 rule” (27, 28), except for the loss of the fourth putative PTHR, all the homologs of PTH and the PTHR genes could be identified in elephant shark (hereafter abbreviated to “e”) (17, 29). PTH, PTHrP, and putative ePTH-L (designated as PTH2 in the references) have already been cloned, while a putative *tip39* gene was identified in the genome database (17, 29). Even though ePTH2 is phylogenetically grouped with other PTH-Ls, the identity of ePTH2 as an ortholog to fish PTH-L was questioned because the gene structure of *ePTH2* is different from teleost *pth-l*, but resembles other *pth* genes. Elephant shark *pth2* has its first intron before the KR cleavage site; whereas teleost *pth-l* genes do not contain this cleavage site, and instead have the first intron before the SRR motif (29). More importantly, ePTH2 is unable to stimulate cAMP production in PTH1R expressing cells, but teleost PTH-L appears to be a potent factor (29).

Regarding the evolution of PTH and the PTHR family in early vertebrates, hypotheses have been proposed largely based on bioinformatics data (17, 21, 22, 30). In lamprey, the distinct phylogenetic positions of the two deduced PTH-like receptors and hormones, suggested a possible scenario of PTHR/PTH evolution before 2WGD (17). PTH1R-like and PTH2R-like genes were produced after the first round of WGD. Then, after the divergence of lamprey, the second round of WGD resulted in only PTH3R due to the lost of the fourth receptor in the PTH2R lineage. For the ligands, PTHrP and TIP39 were proposed to be the pioneers of the PTH family, since they are present in agnathan genomes (18).

In invertebrates, PTHR-like genes are found in the genomes of protostomians, cephalochordates, and urochordates. This indicates the ancestral PTHR was evolved before the deuterostome-protostome split (21, 22, 30). Regarding the ligands, a recent bioinformatics approach has identified PTH-like peptides in tunicates and amphioxi (31). Even though the invertebrate peptides share relatively low homology to the vertebrate PTH family, these observations indicate that the PTH family of peptides already existed in cephalochordates.

## Evolutionary Changes in Gene Structures of the PTH Family

After divergence from their last common ancestor, PTHrP exhibited more changes in gene structure than PTH. The classic gene structure of *pth* contains three exons with the prepro-peptide are encoded in the last two exons. This gene structure is conserved from elephant shark to human (10, 24, 29). However, the *pthrp* gene structure differs through vertebrates by the introduction of exons upstream and/or downstream of the mature peptide coding regions, and presence of splicing variants in *Xenopus*, chicken, and human (10, 32). In addition, unlike PTH and PTH-L, their precursor proteins that lead to single mature peptide, posttranslational processing of mammalian prepro-PTHrP can give rise to three mature peptides: PTHrP, middle region, and osteostatin (33, 34). The increase of *pthrp* gene structure complexity during vertebrate evolution may reflect changes in the physiological roles of this peptide, such as adaptation to the terrestrial environment in tetrapods (9, 35). The new member of the PTH family, PTH-L in non-mammalian vertebrates, was considered as an intermediate between PTH and PTHrP due to its independent phylogenetic position to both PTH and PTHrP (10, 11). Moreover, although the gene structure of PTH-L resembles that of PTH in fugu, chicken, and *Xenopus*, alternative transcripts were reported for *Xenopus* and chicken PTH-L. In addition, all investigated PTH-L peptides contain the “MHD” motif, which is the characteristic of teleost PTHrP (9–11). In elephant shark, all the PTH peptides possess the “MHD” motif (29), suggesting that this motif is present in the common ancestor of PTH peptides and was lost during evolution in some vertebrates. Among all PTH family members, the unique properties of TIP39 are shown by its distant position from other members in phylogenetic analysis, and by the lack of “MHD” or any similar motif (36, 37). The gene structure of TIP39 is highly conserved from mammals to teleosts, and like that of PTH, has three exons (37). The uniqueness of TIP39 could be a result of its earlier divergence from other members of the PTH family.

## Ligand Binding of PTHRs in Vertebrates

Significant levels of sequence homology are found in the first 34 amino acids of mature PTHrP, PTH, and PTH-L peptides. This region is important for receptor binding and receptor activation (9, 10, 16). In mammals, it has been shown that activation of PTH1R by PTH_(1–34)_ and PTHrP_(1–34)_ are comparable to that by the corresponding full length peptides (38, 39). The biological activity of PTH_(1–34)_ was indicated by administration of PTH_(1–34)_ to patients with osteoporosis, which resulted in enhanced bone development (40, 41). PTH_(15–34)_ are required for high affinity binding through interaction with the extracellular N-terminal domain of the receptor (42, 43). Such functional division within PTH in receptor interaction is also likely for PTHrP and TIP39, as well as for interaction of PTH2R with PTH-like peptides, as shown by studies of ligand variants and chimeric receptors (44–47). Functional testing using truncated PTHrP_(2–34, 3–34, and 7–34)_ indicated that these peptides were unable to trigger cAMP synthesis but could stimulate the PLC pathway. In summary, the first few residues are essential to the cAMP pathway but not for receptor binding (48). These properties of truncated PTH_(7–34)_ and PTHrP_(7–36)_ were utilized for developing antagonists of PTH1R (47).

Table 1 summarizes the ligand specificity of the PTHR family in various species. Human (hu) PTH1R can be activated by PTH and PTHrP with indistinguishable potency in downstream signaling pathways, including cAMP, phospholipase C (PLC), and ERK1/2 (49). No activity has been detected for TIP39 in any mammalian PTH1R. It is interesting to note that zebrafish (zf) PTH is able to activate human PTH1R with similar potency to huPTH (24). This indicates the overall structures of the peptides and the receptor binding pockets are highly conserved from mammals to teleosts. Similar ligand specificity is observed in rodents (16, 50) and teleosts (18, 24). In teleosts, fugu (fu) PTHrPA, huPTHrP, and zfPTH exhibit similar efficacies for cAMP activation in zfPTH1R. The only exceptional case is chicken: cPTH1R can be activated by chicken (c) PTHrP as well as by huPTH with similar potency, but to a lower extent by cPTH and cPTH-L (17, 18, 24).

**Table 1:** **Ligand specificity of PTHR family**.

Receptor	Species	Assay	Ligand specificity	Reference
PTH1R	Human	cAMP	hPTHrP ≈ hPTH ≈ zPTH1 ≈ zPTH2 > fPTHrP, no response to TIP39	(14, 24)
		PLC	hPTHrP ≈ hPTH	(19)
		ERK1/2	hPTHrP ≈ hPTH	(51)
		IP1	hPTHrP ≈ hPTH	(51)
		Binding^c^	hPTHrP ≈ hPTH	(52)
	Mouse	cAMP	hPTHrP ≈ hPTH, no response to TIP39	(50)
	Rat	cAMP	rPTHrP ≈ rPTH, no response to TIP39	(16)
	Chicken	cAMP	cPTHrP ≈ hPTH > cPTH-L > cPTH	(17)
		PLC	cPTHrP, slight stimulation by cPTH-L and no response to and cPTH	(17)
	Zebrafish	cAMP	hPTH ≈ hPTHrP ≈ fPTHrPA ≈ zPTH1 > zPTH2	(18, 24)
		PLC	hPTHrP ≈ hPTH	(18)
	Seabream^a^	cAMP	fPTH-L ≈ fPTHrPA > hPTH ≈ bPTH, low respond to hPTHrP, no response to fPTH, and fPTHrPb	(11)
		PLC	fPTHrPA ≈ hPTHrP ≈ hPTH ≈ fPTH-L ≈ fPTHA ≈ fPTHB, no response to fPTHrPB and bPTH	(11)
PTH2R	Human	cAMP	hTIP39 ≈ rTIP39 ≈ rPTH > hPTH	(53, 54)
		PLC	hTIP39 ≈ rTIP39 > rPTH, no response to hPTH	(53)
	Rat	cAMP	hTIP39 > rPTH	(16)
	Zebrafish	cAMP	hTIP39 ≈ zTIP39 > hPTH	(36)
	(SV)	cAMP	zTIP39 > hTIP39	(36)
PTH3R	Chicken	cAMP	cPTHr > cPTH, low response to cPTH-L	(17)
	Zebrafish	cAMP	hPTHrP ≈ fPTHrPA ≈ zPTH1 > zPTH2 > hPTH	(18, 24)
		PLC	no response to hPTHrP and hPTH	(18)
	Seabream^b^	cAMP	fPTHrP, no response to hPTHrP and hPTH	(20)
		PLC	no response to fPTHrP	(20)

*^a^Scale*.

*^b^Enterocyte*.

*^c^Extra-cellular domain*.

*SV, splice variant; h, human; r, rat; z, zebrafish; f, fugu; c, chicken; x, *Xenopus**.

Regarding PTH2R, TIP39 was shown to be the native ligand of PTH2R in mammals and zebrafish (16, 36, 53, 54). huPTH2R responded similarly to TIP39 and PTH in cAMP activation, but PTHs were less effective in PLC activation (53). As was the case for PTH1R, cross-species ligand reactivity is found in zfPTH2R; huTIP39 has similar potency to the endogenous ligand. In contrast, activation of the PLC has not been reported in PTH3R studies. Treatment of zfPTH3R with huPTHrP or PTH did not result in PLC stimulation, and consistent results were observed on treatment of seabream PTH3R with fuPTHrP (18, 20). The ligand bias of PTH3R varies in different species. zfPTH3R shows a preference for fuPTHrP over huPTH, whereas chPTH3R responds similarly to chicken PTHrP and PTH (17, 18). For seabream PTH3R, only fuPTHrPA could activate the receptor (20). In summary, characterization of the PTHR family in various species shows that their ligand specificity is well conserved. Cross-species ligand reactivity of PTHRs demonstrates that PTHRs share similar ligand-receptor binding properties that are conserved throughout vertebrate evolution.

## Functional Changes in the PTH-PTHR System during Vertebrate Evolution

Due to the discovery of the relieving effect of PTH and PTHrP on tetany resulted from parathyroidectomy (12), and also that PTH and PTHrP are related to humoral hypercalcemic syndrome (55–57), initial investigations described the PTH peptides as endocrine hypercalcemic factors. Later research on PTHrP and TIP39 explored other possible physiological functions of these peptides. The vital role of PTHrP in fetal bone development was demonstrated in mouse models by deletion of the PTHrP gene and knockout of PTH1R (58–60). Much work has been undertaken to determine the wider physiological importance of PTHrP and has indicated that PTHrP is a multifunctional paracrine/autocrine factor (61). So far, in placental mammals, accumulated knowledge of the widespread PTH/PTHrP-PTH1R system implicates its pleiotropic functions in regulation of calcium levels including: (1) the control of the release of calcium from bone and increase in renal calcium reabsorption; (2) its effect in the development of bone, cartilage (62–64), pancreas (65, 66), tooth (67–70), and mammary gland (62); and (3) its function to regulate placental calcium supply to the fetus (63, 64, 71). In addition, the C-terminal regions of PTH and PTHrP were postulated to interact with other yet-to-identify receptors, with potential functions remain to be explored (72–74). On the other hand, the expression of PTH2R is restricted to the central nervous system, and the TIP39-PTH2R system is involved in nociceptive signal processing, regulation of hormonal release from the hypothalamus-pituitary axis, and modulation of affective behaviors (44). The distinct and diverse functions of these peptides observed in mammals raised the question of what are the ancestral functions of the PTH-like system in non-mammalian vertebrates and even in invertebrates. Unfortunately, the physiological functions of most PTH peptides in non-mammalian species have not been investigated in depth, and functions of these peptides are largely proposed based on the spatial distribution of their proteins and mRNAs.

The evolution of the parathyroid gland was a key event in the emergence of the tetrapods. Therefore, the expression of PTH in the parathyroid gland was originally linked to an evolutionary concept that the emergence of the PTH-PTHR system was co-evolved with the adaptive transition from calcium-rich aquatic to calcium-deficient terrestrial habitats (9, 75). However, the unexpected identification of PTH in teleosts completely changed this view. Now, the cloning of two PTHs from elephant shark confirms that PTH-like peptides were present far back in evolution as cartilaginous fish, suggesting the original role of these peptides is unrelated to bone formation (29). The endocrine action of PTH released from parathyroid glands in mammals could not be observed in fish, which have no parathyroid gland. Instead, it was suggested that PTH is a paracrine factor in non-mammalian vertebrates such as fish and *Xenopus* (10). Although the function of PTH in fish remains poorly described, PTH-L which is absent from placental mammals, was proposed to mimic the role of mammalian PTH in fish, since fuPTH-L was found to be a potent factor causing whole body calcium influx in seabream larvae but no response was detected using fugu PTHA or PTHB (11). Based on the recent characterization of PTH-L from chicken and *Xenopus*, it was hypothesized that there was a functional transition between PTH-L and PTH during vertebrate evolution. In this scenario, PTH-L gradually lost its calcitropic activity and eventually was lost in mammalian genomes, while PTH replaced PTH-L in mammals to become the main endocrine regulator of calcium with expression restricted only to the parathyroid gland (10). One piece of supporting evidence for such a transition is that PTH-L and PTHrP in chicken and *Xenopus* show overlapping tissue distributions, indicating redundancy. Similarly, immunohistochemistry in elephant shark showed that the PTH orthologs, PTH1 and PTH2, exhibit widespread localization and considerable locational overlap with PTHrP (29). This implies that PTH-like peptides in non-mammalian vertebrates and cartilaginous fish may partially share their physiological role(s) with PTHrP. However, there is a mismatch between the results of widespread protein detection and restricted mRNA expression of elephant shark PTH1 and PTH2 (29). Whether this reflects the endocrine action of the two peptides remains to be determined.

Phylogenetic analysis of two predicted PTH-like in the lamprey genome, TIP39 and PTHrP, suggests that these are probably the ancestral members of the PTH peptide family found in vertebrates (17). In general, the expression of these two peptides appears to be unchanged in the vertebrate lineage (76). Unfortunately, no experiments have been conducted to determine expression patterns of lamprey TIP39. Wide tissue expression of PTHrP was observed in all investigated species to date, and its detection in skin, skeletal and, cardiac muscle, and kidney is conserved from lamprey to human (9, 29, 77, 78). The very similar tissue expression patterns of TIP39 and PTHrP in zebrafish and lamprey respectively indicate that TIP39 and PTHrP possess ancestral functions compared with PTH and PTH-L. In invertebrates, use of heterologous antisera in immunohistochemistry enabled detection of PTH-like peptides in snail, cockroach, and amphioxus neural tissue (79). This indicates that the origin of the PTH family may be far earlier in evolution than our expectation.

## Conclusion

Based on bioinformatics, the presence of PTHR dates back to an ancestor before the deuterostome-protostome split. Although the true identity of PTH-like peptides in invertebrates requires clarification, the peptide family likely co-evolved with its cognate receptors in vertebrates since agnatha. Duplication of this ancestral PTHR through 2WGD resulted in the PTHR family found in modern jawed vertebrate species. Insight from the recent characterization of PTH and the PTHR family from non-mammalian species, and the discovery of putative PTHR-like and PTH-like, in a lamprey genome, reveals that the pioneer of the PTH-PTHR system, and its physiological properties, are likely fundamentally conserved throughout vertebrate evolution.

## Conflict of Interest Statement

The authors declare that the research was conducted in the absence of any commercial or financial relationships that could be construed as a potential conflict of interest. The Associate Editor Hubert Vaudry declares that, despite having collaborated with author Billy K. C. Chow, the review process was handled objectively and no conflict of interest exists.
